# Transcriptome Analysis of mRNAs and Long Non-Coding RNAs During Subsequent Embryo Development of Porcine Cloned Zygotes After Vitrification

**DOI:** 10.3389/fgene.2021.753327

**Published:** 2021-12-17

**Authors:** Decai Xiang, Baoyu Jia, Jianxiong Guo, Qingyong Shao, Qionghua Hong, Hongjiang Wei, Guobo Quan, Guoquan Wu

**Affiliations:** ^1^ Yunnan Provincial Genebank of Livestock and Poultry Genetic Resources, Yunnan Provincial Engineering Laboratory of Animal Genetic Resource Conservation and Germplasm Enhancement, Yunnan Animal Science and Veterinary Institute, Kunming, China; ^2^ Key Laboratory of Animal Gene Editing and Animal Cloning in Yunnan Province, College of Veterinary Medicine, Yunnan Agricultural University, Kunming, China

**Keywords:** pig, cloned zygote, vitrification, transcriptome, long non-coding RNA

## Abstract

Cryopreservation of porcine cloned zygotes has important implications for biotechnology and biomedicine research; however, lower embryo developmental potential remains an urgent problem to be resolved. For exploring the sublethal cryodamages during embryo development, this study was designed to acquire the mRNA and long non-coding RNA (lncRNA) profiles of 2-cells, 4-cells and blastocysts derived from vitrified porcine cloned zygotes using transcriptome sequencing. We identified 167 differentially expressed (DE) mRNAs and 516 DE lncRNAs in 2-cell stage, 469 DE mRNAs and 565 lncRNAs in 4-cell stage, and 389 DE mRNAs and 816 DE lncRNAs in blastocyst stage. Functional enrichment analysis revealed that the DE mRNAs during embryo development were involved in many regulatory mechanisms related to cell cycle, cell proliferation, apoptosis, metabolism and others. Moreover, the target genes of DE lncRNAs in the three embryonic stages were also enriched in many key GO terms or pathways such as “defense response”, “linoleic acid metabolic process”, “embryonic axis specification”, “negative regulation of protein neddylation”, etc., In conclusion, the present study provided comprehensive transcriptomic data about mRNAs and lncRNAs for the vitrified porcine cloned zygotes during different developmental stages, which contributed to further understand the potential cryodamage mechanisms responsible for impaired embryo development.

## Introduction

In addition to being important livestock, pigs are also ideal large animal models for biological and biomedical research because they share similar anatomy, genetics, physiology, and pathology with humans (Tang et al., 2007; Wilkinson et al., 2013). The generation of genetically modified pigs has great promise in livestock industry and human disease treatment including regenerative medicine, xenotransplantation, stem cell therapy and others (Yang and Wu, 2018; Yue et al., 2021). These special pigs can be produced through the combination of gene editing technology and cloning by somatic cell nuclear transfer (SCNT) (Whitelaw et al., 2016). Furthermore, the cloned embryos obtained using these technical procedures are indispensable to result in genetically identical offspring (Keefer, 2015). For research and therapeutic purposes, the production of embryonic stem cells with the same genetic characteristics also requires cloned embryos by SCNT technique (Gouveia et al., 2020). Therefore, it is very essential to effectively cryopreserve porcine cloned embryos in order to genetic resource preservation as well as convenient research and utilization in related fields.

Cryopreservation of porcine embryos has been consistently considered difficult to perform owing to high chilling and freezing sensitivity caused by abundant cytoplasmic lipid droplets (Jin et al., 2013). With the development of vitrification techniques, the cryosurvival efficiency of porcine embryos at various developmental stages has been greatly improved in recent years (Wu et al., 2016; Bartolac et al., 2018; Nguyen et al., 2018; Nohalez et al., 2018; Xiang et al., 2019), and live piglets are also produced from the vitrified embryos derived *in vivo* or *in vitro* after transfer into recipients (Beebe et al., 2011; Misumi et al., 2013; Mito et al., 2015; Cuello et al., 2016; Kamoshita et al., 2017). However, vitrification results in the sublethal damages of surviving embryos as well, which may affect the subsequent properties for embryonic development such as blastocyst formation and embryo implantation (Tajima et al., 2020; Vining et al., 2021). Some studies have observed that vitrification of porcine early embryos including zygotes and 2- and 4- cells reduces their potential to develop to the blastocyst stage (Esaki et al., 2004; Somfai et al., 2009; Gomis et al., 2013). Although not reported frequently, there are some studies being conducted on the vitrification of porcine cloned embryos, resulting in an acceptable survival rate (Du et al., 2007; Kawakami et al., 2008; Nakano et al., 2011; Jia et al., 2020). Moreover, porcine cloned embryos may be appropriate for vitrifying at very early developmental stages such as the zygote stage, so they can be well applied to embryo transfer or mechanism research relate to embryonic development. Although vitrification is proved to not significantly reduce survival rate, the porcine cloned zygotes still show lower blastocyst formation rate as compared with their fresh counterparts (27.1 vs. 36.7%) (Jia et al., 2020). Thus it is very necessary to explore the structural and functional changes that occur during embryonic development of vitrified porcine cloned zygotes.

In recent years, increased focus has been placed on detecting the variation in gene expression for oocytes and embryos after vitrification. It is an effective means to analyze differences of important genes between fresh and vitrified embryos by using quantitative real-time PCR (qRT-PCR). For instance, vitrification of porcine blastocysts has been reported to result in altered expression of *POU5F1* and *HSPA1A* (Castillo-Martín et al., 2015). In our previous study, the resultant blastocysts from vitrified early-stage porcine cloned embryos showed increased expression of *PCNA*, *CPT1*, *POU5F1* and *DNMT3B* (Jia et al., 2020). Moreover, a microarray approach has also been applied to analyze the impact of vitrification on transcriptome profile of in *vivo*-derived porcine blastocysts (Cuello et al., 2021). On the other hand, the Smart-seq2 protocol has evolved into widely used tools for comprehensive analysis of the differential transcriptomes for vitrified oocytes in several mammalian species such as mice (Gao et al., 2017), pigs (Jia et al., 2019) and cattle (Wang et al., 2017; Huang et al., 2018; Zhang F. et al., 2020), which allows amplification to be performed for a small sample prior to the RNA sequencing (RNA-seq) (Picelli et al., 2014). More recently, the transcriptome features and differences of vitrified porcine cloned blastocysts have been investigated using RNA-seq (Zhang L. et al., 2020).

Long non-coding RNAs (lncRNAs) are important members of non-coding RNAs family, with transcripts longer more than 200 nucleotides. Accumulating evidences indicate that lncRNAs play assorted functions in the field of reproduction, such as spermatogenesis (Trovero et al., 2020), follicle growth (Ernst et al., 2018), oocyte maturation (Jiao et al., 2020; Wang J. et al., 2020; Wang J. J. et al., 2020; Yang et al., 2021), oocyte-to-embryo transition (Svoboda, 2017), embryonic development (Bouckenheimer et al., 2016) and others. Recent researches confirm that epigenetic modification can also be regulated by lncRNAs in oocytes and SCNT embryos (Yang et al., 2020; Deng et al., 2021). Based on above description, the present study applied the RNA-seq technique coupled with Smart-seq2 method to obtain the mRNA and lncRNA profiles of 2-cells, 4-cells and blastocysts derived from vitrified porcine cloned zygotes, which will directly lead to a deeper understanding of the molecular mechanism responsible for embryonic developmental impairment.

## Materials and Methods

### Chemicals and Reagents

In this study, all chemicals and reagents were purchased from Sigma-Aldrich Chemical Company (Shanghai, China), unless otherwise specified. Tissue culture medium-199 (TCM-199), Dulbecco’s phosphate buffered saline (DPBS) and knockout serum replacement were obtained from ThermoFisher Scientific (Shanghai, China).

### Oocyte Collection and Maturation *in vitro*

Porcine pre-pubertal ovaries were obtained from a local slaughterhouse and transported to the laboratory within 2 h in saline at 35–37°C. There, follicular fluids were aspirated from 3 to 8 mm antral follicles using a 20 ml-disposable syringe with an 18-gauge needle. After settlement for a short time, the sediments were resuspended two times with Tyrode’s lactate-HEPES-polyvinyl alcohol (TLH-PVA) medium (Funahashi et al., 1997) and then observed under a stereomicroscope (Olympus, Tokyo, Japan). Cumulus-oocyte complexes (COCs) with homogeneous ooplasm and three or more layers of compact cumulus cells were selected for *in vitro* maturation (IVM). In brief, groups of 50–70 COCs were washed three times in IVM medium and then cultured in each well of a 24-well plate (Costar, Corning, NY, United States) containing 500 μL IVM medium for 42–44 h at 39°C in an atmosphere of 5% CO_2_ with saturated humidity. The IVM medium consisted of TCM-199 supplemented with 3.05 mM D-glucose, 0.57 mM cysteine, 0.91 mM sodium pyruvate, 10% (v/v) porcine follicular fluid, 10 ng/ml epidermal growth factor, 0.5 μg/ml follicle-stimulating hormone and 0.5 μg/ml luteinizing hormone. At the end of IVM, COCs were denuded of cumulus cells by repeatedly pipetting in TLH-PVA medium containing 0.1% (w/v) hyaluronidase. Oocytes with an extruded first polar body and evenly granuled cytoplasm were selected for enucleation.

### Donor Cell Preparation

The donor cells were isolated from the ear tissues of Diannan miniature pigs and then cultured in DMEM supplemented with 10% fetal bovine serum (Ausbian, Sydney, Australia) at 38.5°C in a humidified atmosphere of 5% CO_2_. After four to eight passages, donor cells were induced entry into the G0/G1 phase and then harvested with trypsin for SCNT.

### SCNT and Embryo Culture

The procedures of SCNT were carried out as described previously (Wei et al., 2013). Briefly, oocytes were incubated in porcine zygote medium-3 (PZM-3) (Yoshioka et al., 2002) supplemented with 0.1 μg/ml demecolcine and 0.05 M sucrose for 0.5–1 h. Moreover, the first polar body and chromosomes were enucleated using an aspiration glass pipette in TLH-PVA medium containing 0.1 μg/ml demecolcine and 5 μg/ml cytochalasin B. A donor cell was inserted into the perivitelline space of an enucleated oocyte. The oocyte–donor cell couplets were placed between two wires of a fusion chamber overlaid with fusion medium (0.28 M mannitol, 0.1 mM MgSO_4_ and 0.5 mM HEPES), and then fused by a direct current (DC) pulse of 200V/mm for 20 μs using an Electro Cell Fusion Generator (LF201, NEPA GENE Co., Ltd. Japan). After incubating in PZM-3 for 0.5–1 h, they were activated by a DC pulse of 150 V/mm for 100 μs in activation medium (0.28 M mannitol, 0.1 mM MgSO_4_, 0.05 mM CaCl_2_ and 0.5 mM HEPES), followed by a 2–4 h culture in PZM-3 supplemented with 5 μg/ml cytochalasin B at 39°C under a humidified atmosphere of 5% CO_2_ in air. Finally, reconstructed cloned embryos were washed three times in PZM-3 and then cultured in the same medium under conditions described above.

### Vitrification and Warming

According to our previous study (Jia et al., 2020), the presumptive cloned zygotes after chemical activation were submitted to vitrification and warming. Basic medium (BM) was composed of DPBS with 20% (v/v) knockout serum replacement. Laboratory temperature was maintained at 25 ± 1°C. After washing in BM for a moment, the zygotes were placed into equilibration solution supplemented with 15% (v/v) ethylene glycol (EG) for 3 min, and then transferred to vitrification solution consisting of 0.6 M sucrose, 50 mg/ml polyvinylpyrrolidone and 35% (v/v) EG for 20–30 s. Thereafter, a group of about 10 zygotes were loaded onto a Cryotop carrier (Kitazato Biopharma, Shizuoka, Japan) with minimal vitrification solution and immediately plunged into liquid nitrogen (LN_2_) for long term storage. Warming was performed on a 42°C hot plate by directly dipping the tip of Cryotop carrier into 1.0 M sucrose for 1 min at 42°C. The vitrified zygotes were sequentially transferred stepwise into 0.5 and 0.25 M sucrose for 2.5 min at 39°C, respectively. Following incubation in BM for 5 min, they were washed three times in PZM-3 and subjected to *in vitro* culture.

### Sample Collection and RNA-Seq

Three biological replicates were performed for all experiments. For the experimental groups, the cloned zygotes were vitrified and then cultured in PZM-3 to develop into 2-cells, 4-cells and blastocysts. The fresh cloned embryos that developed to the 2-cell, 4-cell and blastocyst stages were used as the corresponding non-vitrified controls. The cloned embryos of each stage during preimplantation development were collected at the following time points after chemical activation: 24 h for 2-cells (4 per pool), 48 h for 4-cells (2 per pool), and 144 h for blastocysts (1 per pool). These embryos were transferred into cell lysis buffer, immediately frozen in LN_2_ and stored at −80°C. The full-length cDNA was generated with SMART-Seq v4 Ultra Low Input RNA Kit (Takara Bio United States, Mountain View, CA. United States) following the manufacturer’s instructions. Briefly, first-strand cDNA was synthesized using 3′ SMART-Seq CDS Primer II A and SMART-Seq v4 Oligonucleotide, following by cDNA amplification through LD-PCR. Then the amplified cDNA was purified by immobilization on AMPure XP beads and validated using the Agilent High Sensitivity DNA Kit on an Agilent 2100 bioanalyzer (Agilent Technologies, CA, United States). Library preparation was generated from an equal amount of cDNA samples using the Illumina Nextera XT DNA Sample Preparation Kit (Illumina, San Diego, CA, United States), and library quality was assessed in an Agilent 2100 bioanalyzer. RNA-Seq was conducted on the Illumina Novaseq™ 6000 platform (Illumina, San Diego, CA, United States) in the 150 bp paired-end configuration by LC-Bio Sciences (Hangzhou, China).

### Transcripts Assembly

Firstly, the reads that contained adaptor contamination, low quality bases and undetermined bases were removed by Cutadapt (https://code.google.com/p/cutadapt/). Then FastQC (http://www.bioinformatics.babraham.ac.uk/projects/fastqc/) was used to verify sequence quality. The clean reads were mapped to the genome data of *Sus scrofa* with Bowtie2 (http://bowtie-bio.sourceforge.net/bowtie2/index.shtml) and Hisat2 (https://daehwankimlab.github.io/hisat2/), and mapped reads of each sample were subsequently assembled using StringTie (http://ccb.jhu.edu/software/stringtie/). Finally, all transcriptomes were merged to reconstruct a comprehensive transcriptome using a Perl script.

### LncRNA Identification

Transcripts overlapping with known mRNAs and transcripts shorter than 200 bp were discarded. Then we predicted transcripts with coding potential by utilizing coding potential calculator (CPC, http://cpc.cbi.pku.edu.cn/) and coding-non-coding index (CNCI, http://cpc.cbi.pku.edu.cn/) All transcripts with score CPC < −1 and score CNCI <0 were removed. The remaining transcripts were considered as lncRNAs.

### Different Expression Analysis of mRNAs and lncRNAs

StringTie was used to perform expression level analysis for mRNAs and lncRNAs by calculating fragments per kilobase of exon per million fragments. The differentially expressed (DE) mRNAs and lncRNAs were determined with the threshold of |log_2_ (fold change)| > 1 and statistical significance (*p*-value < 0.05) by R package-edgeR.

### Target Gene Prediction

To explore the function of lncRNAs, we predicted their target genes in cis, as lncRNAs may play a cis role to act on neighboring target genes. In the present study, coding genes within 100 Kb upstream and downstream of DE lncRNAs on the genome were selected by a python script. Only the DE mRNAs were considered as potential cis-target genes in order to reduce false positives.

### Functional Enrichment Analysis

GO enrichment analysis was carried out for all DE mRNAs and cis-target genes of DE lncRNAs using BLAST2GO (https://www.blast2go.com/), according to cellular component, biological process and molecular function. Meanwhile, the KEGG pathway database (http://www.genome.jp/kegg/) was used to perform pathway enrichment analysis. GO terms and KEGG pathways with *p*-value < 0.05 were considered significantly enriched.

### qRT-PCR

Total complementary DNA (cDNA) was isolated from 2-cells (10 per pool), 4-cells (5 per pool) and blastocysts (5 per pool) using a TransScript®-Uni Cell to cDNA Synthesis SuperMix for qPCR (TransGen Biotech, Beijing, China) according to the manufacturer’s protocol, and stored at −20°C until used. qRT-PCR was conducted using a CFX Real-Time PCR Detection System (Bio-Rad, Hercules, CA, United States) with the Fast qPCR Mix (Tsingke, Beijing, China). Reaction conditions were 95°C for 1 min, followed by 40 cycles of 95°C for 10 s and 60°C for 15 s. Four replicates were made for each reaction. qRT-PCR was performed in three independent biological replicates for each sample. Relative expression of selected genes was calculated by the 2^−ΔΔCT^ method after normalization to GAPDH. Primer sequences used in this experiment are listed in Supplementary Table S1.

## Results

### Overview of RNA-Seq Data

In the present study, a total of 18 cDNA libraries were constructed using 2-cells, 4-cells and blastocysts derived from fresh and vitrified porcine cloned zygotes. We obtained 146.81 Gb raw data after sequencing by the Illumina Novaseq™ 6000 platform. After quality control, 110.88 Gb valid date were retained, with an average Q30 ratio of 98.59% and GC contents of 47–54%. Moreover, 75.81–90.17% of valid reads were aligned against the *Sus scrofa* reference genome, and the unique mapping ratio were 57.50–69.96%. The detailed information about data quality and mapping statistics are shown in Supplementary Table S2.

### Identification of lncRNAs

**FIGURE 1:**
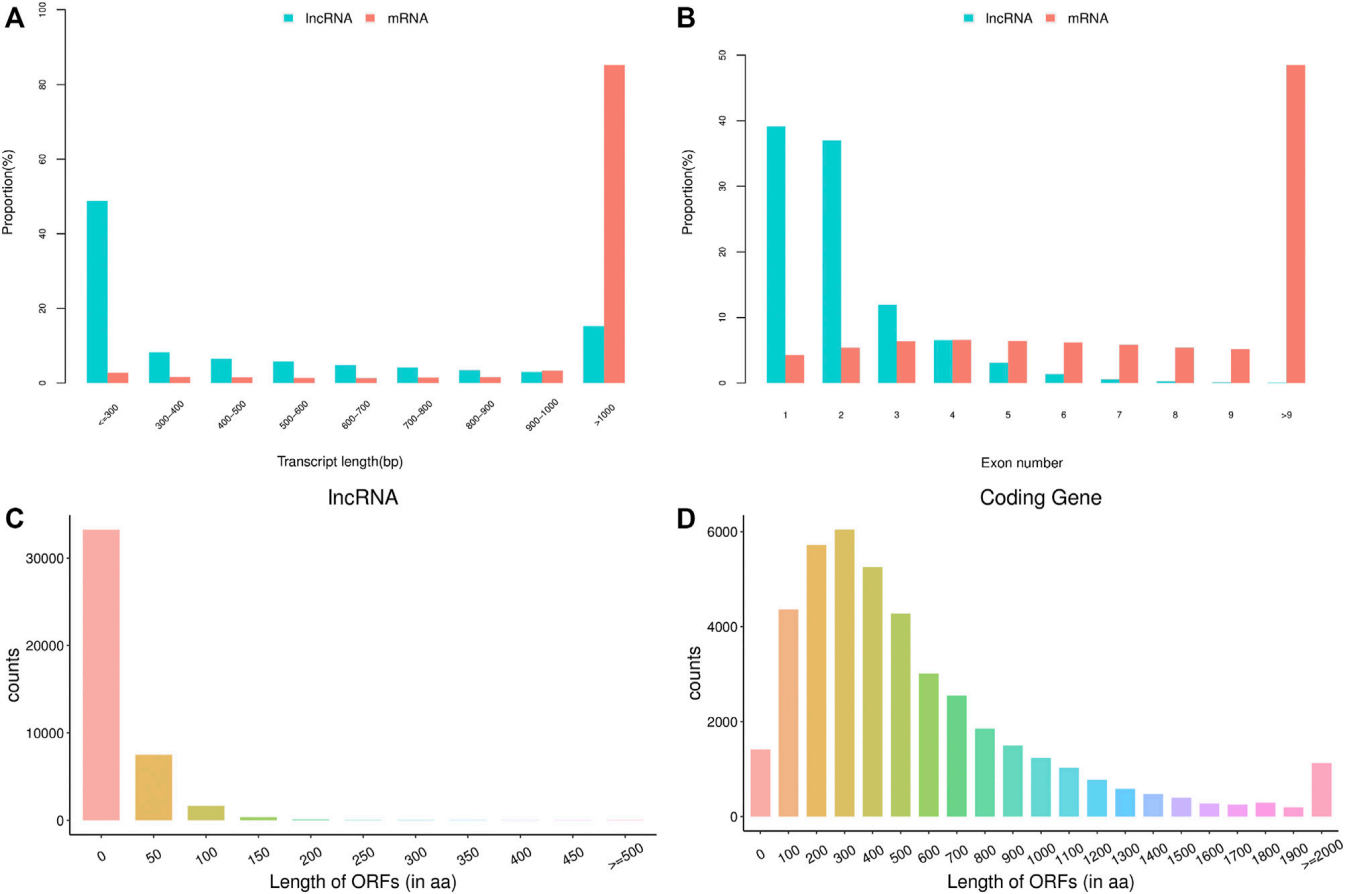
The basic features of predicted long non-coding RNAs (lncRNAs) and mRNAs. **(A)** transcript length of lncRNAs and mRNAs, **(B)** exon number of lncRNAs and mRNAs, **(C)** open reading frame (ORF) length of lncRNAs, **(D)** ORF length of mRNAs.

The basic features of lncRNAs were identified and compared with mRNAs by analyzing the transcript length, exon number and open reading frame (ORF) length (Figure 1). Transcript length of lncRNAs was mainly between 200 and 1,000 bp, while most of mRNA transcripts showed more than 1,000 bp length. The majority of lncRNAs consisted of one or two exon(s), which had fewer exon number compared with mRNAs. Moreover, ORF length of lncRNAs was mostly distributed within 0–100 aa, and was also shorter than that of mRNAs. The above data in this study conformed to the typical characteristics of lncRNAs. In addition, a total of 140420 lncRNAs were identified from the 18 libraries, including 401 known lncRNAs and 140019 novel lncRNAs.

### Differential Expression Analysis of mRNAs and lncRNAs

**FIGURE 2:**
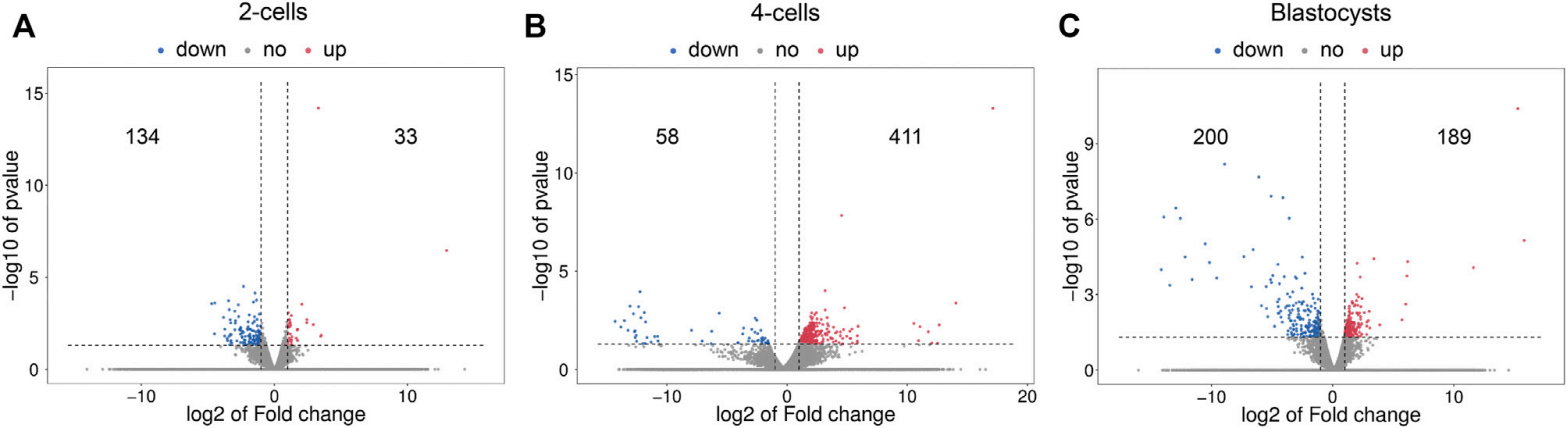
Volcano plots of differentially expressed mRNAs in **(A)** 2-cells, **(B)** 4-cells and **(C)** blastocysts derived from vitrified porcine cloned zygotes. Red points indicate significant up-regulated mRNAs, blue points indicate significant down-regulated mRNAs, while grey points indicate mRNAs without differences. The X-axis represents the log2 fold change, Y-axis means the −log10 (*p*-value).

The volcano plots of the differential mRNA expression analysis are revealed in Figure 2. For the comparison of 2-cells from vitrified and fresh zygotes, a total of 167 DE mRNAs were found of which 33 were up-regulated and 134 were down-regulated (Supplementary Table S3). Moreover, we found that 469 mRNAs were differentially expressed in the 4-cells from vitrified zygotes, including 411 up-regulated and 58 down-regulated mRNAs (Supplementary Table S4). Comparing vitrified zygote-derived blastocysts with fresh blastocysts, there were189 up-regulated and 200 down-regulated DE mRNAs (Supplementary Table S5).

**FIGURE 3:**
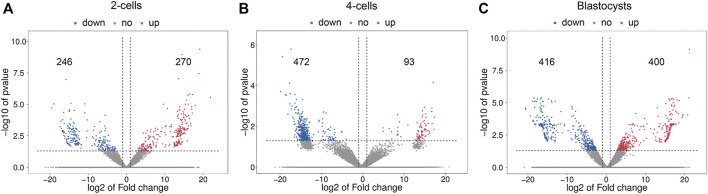
Volcano plots of differentially expressed lncRNAs in **(A)** 2-cells, **(B)** 4-cells and **(C)** blastocysts derived from vitrified porcine cloned zygotes. Red points indicate significant up-regulated lncRNAs, blue points indicate significant down-regulated lncRNAs, while grey points indicate lncRNAs without differences. The X-axis represents the log2 fold change, Y-axis means the −log10 (*p*-value).

Figure 3 shows the differential lncRNA abundances as volcano plots. The 2-cells from vitrified and fresh zygotes were found to present 516 DE lncRNAs, with 270 up-regulated and 246 down-regulated lncRNAs (Supplementary Table S6). Moreover, the 4-cells from vitrified zygotes had 565 lncRNAs with differential expression including 93 up-regulated and 472 down-regulated lncRNAs compared with the fresh 4-cells (Supplementary Table S7). There were a total of 816 DE lncRNAs (400 up-regulated and 416 down-regulated) between vitrified zygote-derived blastocysts and fresh blastocysts (Supplementary Table S8).

**FIGURE 4:**
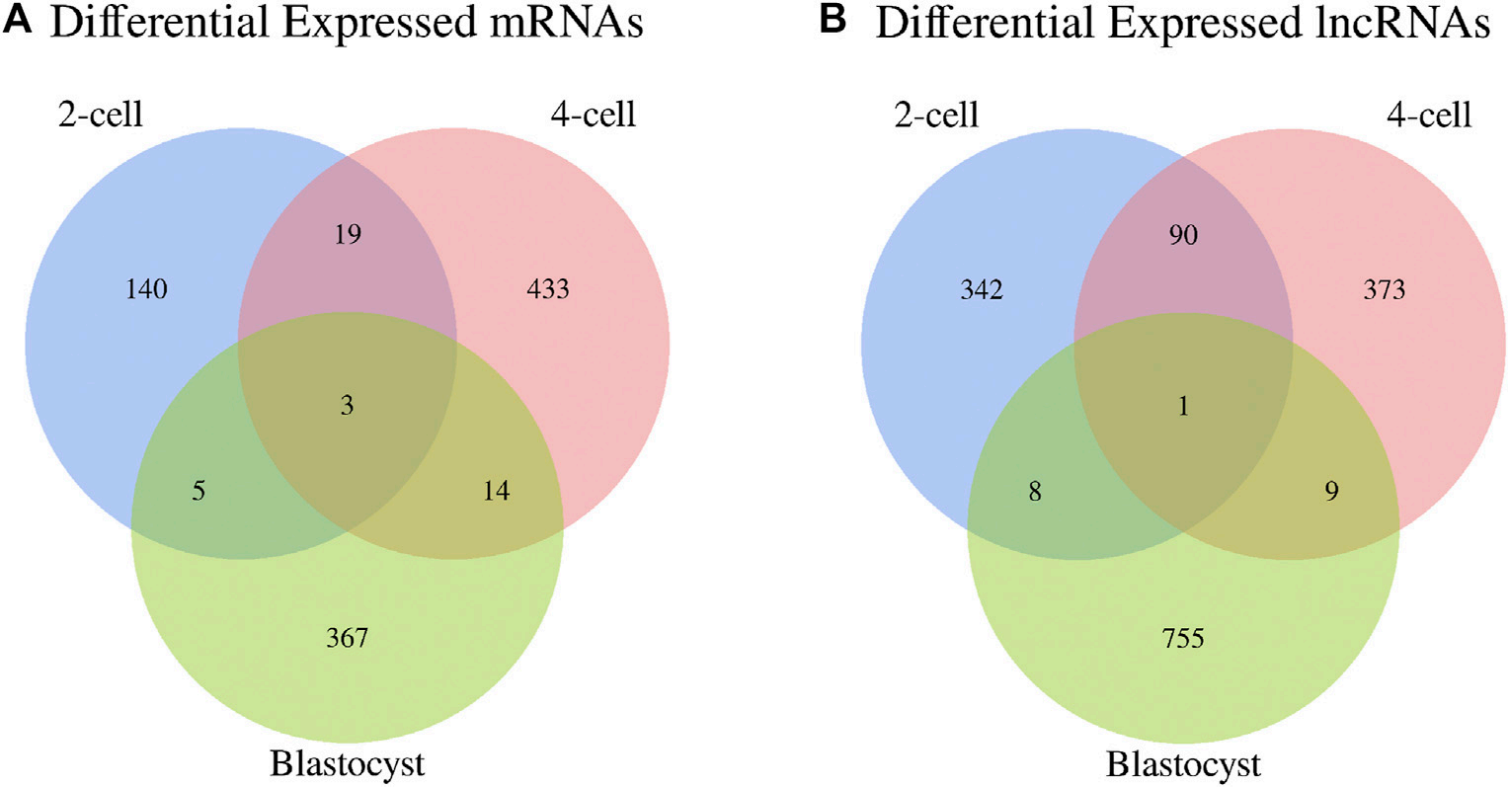
Venn diagrams of differentially expressed **(A)** mRNAs and **(B)** lncRNAs among three libraries.

In addition, the Venn diagrams of mRNAs and lncRNAs were constructed from three comparisons in order to further analyze the intersection genes. We found that the shared DE mRNAs were 3 in the development stages of 2-cell, 4-cell and blastocyst (Figure 4A). Moreover, there was only 1 overlapping lncRNA for these three embryonic stages (Figure 4B).

Finally, a cis-target gene prediction was performed to obtain the potential target mRNAs of the DE lncRNAs (Supplementary Table S9). 15 pairs of lncRNA-mRNA were identified in 2-cells from vitrified and fresh zygotes, and the target genes contained *SMARCAD1*, *MYSM1*, *POGZ*, *ACIN1*, *GIN1*, *SLC31A1*, *LARP7*, *FAR1*, *NNT*, *TXNL4B* and *MYEF2*. Comparing 4-cells from vitrified zygotes with control, there were 4 pairs of lncRNA-mRNA, with target genes of *ITPKA*, *NDEL1*, *RELCH* and *DAPL1*. Moreover, a total of 53 pairs of lncRNA-mRNA were acquired between vitrified zygote-derived blastocysts and fresh blastocysts. Some important genes were found, such as *ABCF3*, *CCAR1*, *GDF9*, *ISYNA1*, *KIF20B* and so on.

### Functional Enrichment Analysis of mRNAs and lncRNA Targets in 2-Cells

**FIGURE 5:**
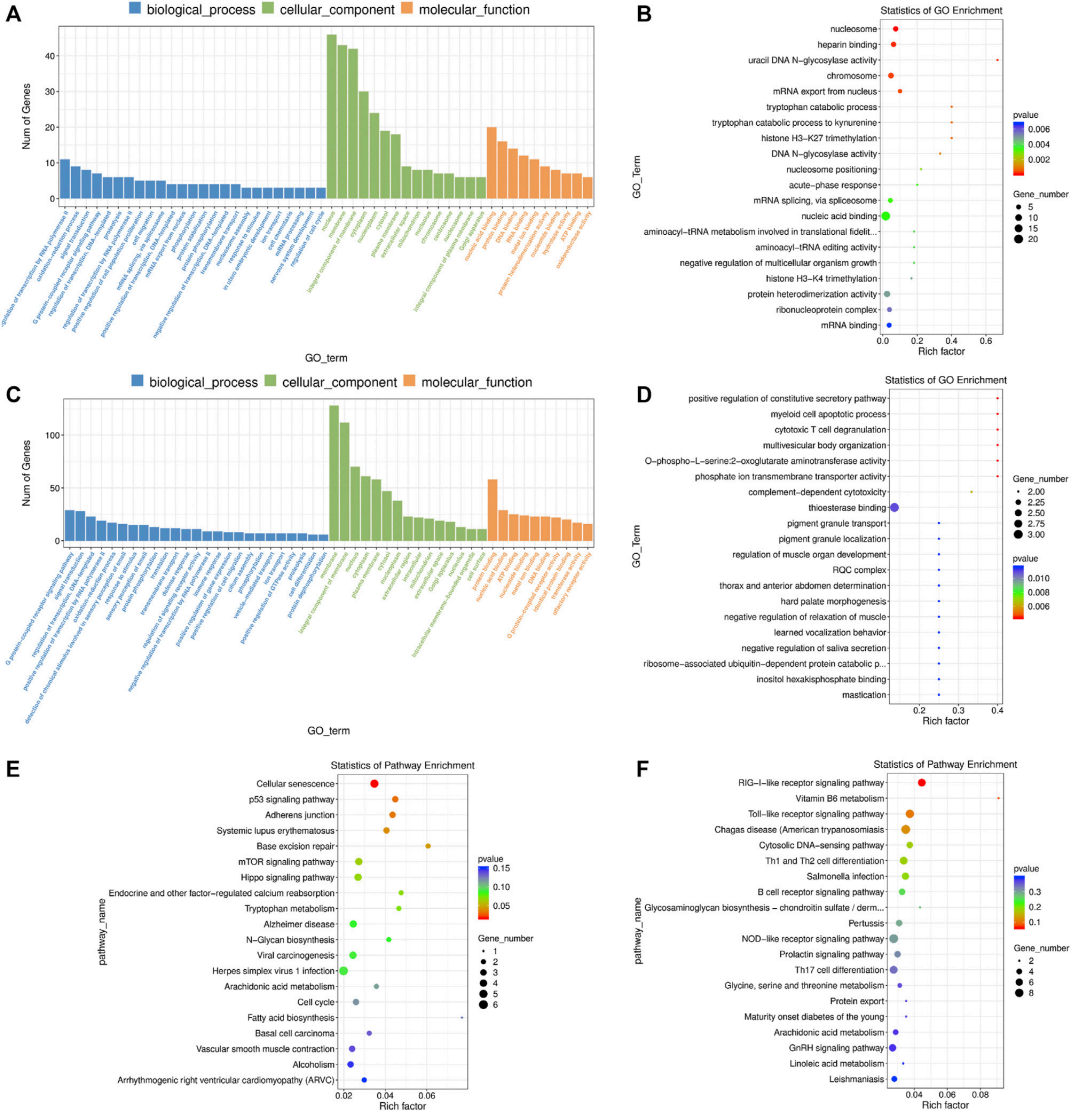
GO and KEGG enrichment analysis of differentially expressed (DE) mRNAs and lncRNA targets in 2-cells derived from vitrified porcine cloned zygotes. **(A)** Histogram and **(B)** scatter plot of GO enrichment for DE mRNAs; **(C)** histogram and **(D)** scatter plot of GO enrichment for target genes of DE lncRNAs; **(E)** scatter plot of KEGG enrichment for DE mRNAs; **(F)** scatter plot of KEGG enrichment for target genes of DE lncRNAs.

Firstly, we performed a GO enrichment analysis for these DE mRNAs (Figures 5A,B and Supplementary Table S10). The enriched GO terms were primarily associated with “nucleus”, “nucleic acid binding”, “negative regulation of transcription by RNA polymerase II”, “protein heterodimerization activity”, “oxidation-reduction process”, “cell migration”, “nucleosome assembly”, “regulation of cell cycle”, “glutathione metabolic process”, “negative regulation of TOR signaling”, etc., In addition, the DE mRNAs were enriched in several important KEGG pathways including “cellular senescence”, “mTOR signaling pathway”, “hippo signaling pathway”, “p53 signaling pathway” and “adherens junction” (Figure 5E and Supplementary Table S10).

For the DE lncRNAs, their target genes were mainly involved in the GO terms of “defense response”, “cellular response to amino acid starvation”, “thioesterase binding”, “MAP kinase activity”, “calcium ion regulated exocytosis”, “L-serine biosynthetic process”, “positive regulation of reactive oxygen species biosynthetic process”, “cellular phosphate ion homeostasis”, “linoleic acid metabolic process” and others (Figures 5C,D and Supplementary Table S11). Moreover, KEGG pathway results showed that the target genes of lncRNAs were involved in “RIG-I-like receptor signaling pathway” and “Vitamin B6 metabolism” (Figure 5F and Supplementary Table S11).

### Functional Enrichment Analysis of mRNAs and lncRNA Targets in 4-Cells

**FIGURE 6:**
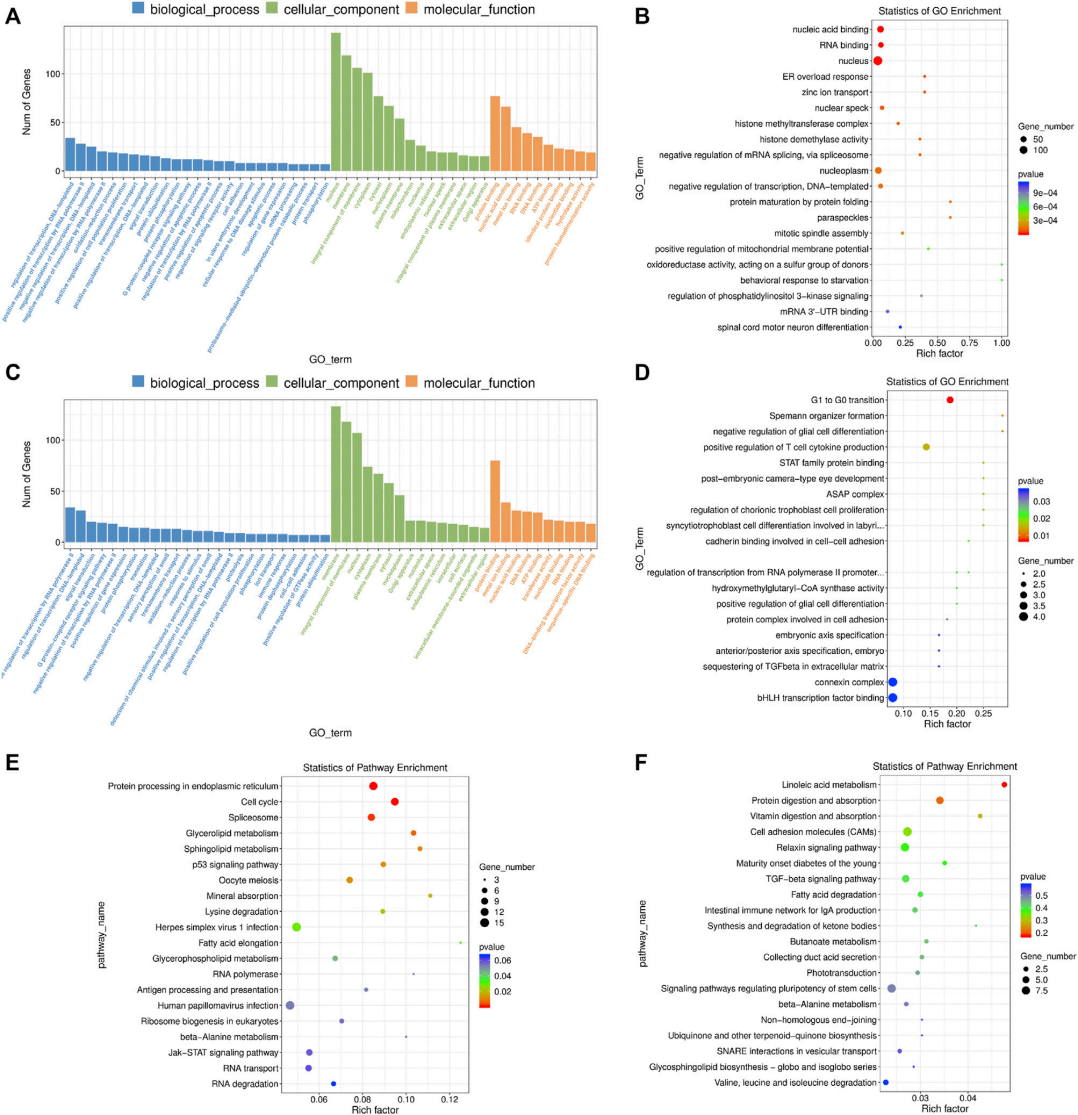
GO and KEGG enrichment analysis of differentially expressed (DE) mRNAs and lncRNA targets in 4-cells derived from vitrified porcine cloned zygotes. **(A)** Histogram and **(B)** scatter plot of GO enrichment for DE mRNAs; **(C)** histogram and **(D)** scatter plot of GO enrichment for target genes of DE lncRNAs; **(E)** scatter plot of KEGG enrichment for DE mRNAs; **(F)** scatter plot of KEGG enrichment for target genes of DE lncRNAs.

We found that the DE mRNAs were enriched in large numbers of significant GO terms such as “negative regulation of transcription, DNA-templated”, “positive regulation of cell population proliferation”, “protein ubiquitination”, “positive regulation of apoptotic process”, “histone methyltransferase complex”, “response to oxidative stress”, “mitotic cell cycle”, “response to endoplasmic reticulum stress”, “autophagy” and “cell redox homeostasis” (Figures 6A,B and Supplementary Table S12). The enriched KEGG pathways had “cell cycle”, “spliceosome”, “p53 signaling pathway”, and multiple metabolic pathways including “glycerolipid metabolism”, “sphingolipid metabolism”, “glycerophospholipid metabolism” and “beta-alanine metabolism” (Figure 6E and Supplementary Table S12).

Based on GO enrichment analysis, the target genes of DE lncRNAs were major in “connexin complex”, “G1 to G0 transition”, “histone H3-K4 trimethylation”, “mitotic chromosome condensation”, “mitochondrial respiratory chain complex IV assembly”, “embryonic axis specification”, “cadherin binding involved in cell-cell adhesion”, “linoleic acid metabolic process” and so on (Figures 6C,D and Supplementary Table S13). In addition, KEGG pathways focused on “linoleic acid metabolism”, “cell adhesion molecules”, “TGF-beta signaling pathway”, “fatty acid degradation” and “signaling pathways regulating pluripotency of stem cells” (Figure 6F and Supplementary Table S13).

### Functional Enrichment Analysis of mRNAs and lncRNA Targets in Blastocysts

**FIGURE 7:**
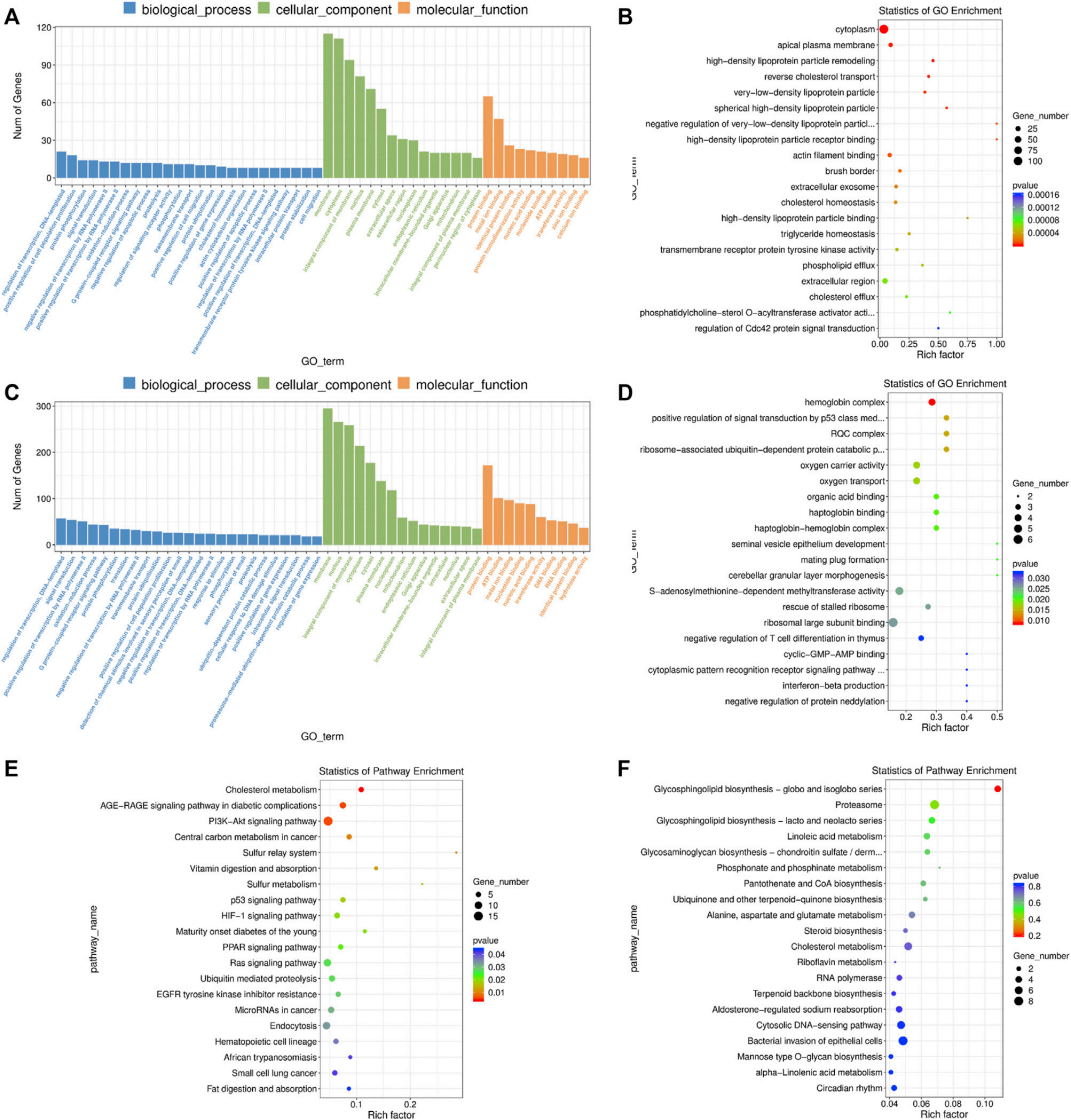
GO and KEGG enrichment analysis of differentially expressed (DE) mRNAs and lncRNA targets in blastocysts developed from vitrified porcine cloned zygotes. **(A)** Histogram and **(B)** scatter plot of GO enrichment for DE mRNAs; **(C)** histogram and (D) scatter plot of GO enrichment for target genes of DE lncRNAs; **(E)** scatter plot of KEGG enrichment for DE mRNAs; **(F)** scatter plot of KEGG enrichment for target genes of DE lncRNAs.

The DE mRNAs were significantly enriched to GO terms, including “metal ion binding”, “positive regulation of cell population proliferation”, “negative regulation of apoptotic process”, “positive regulation of cell migration”, “actin cytoskeleton organization”, “positive regulation of ERK1 and ERK2 cascade”, “cell-cell junction”, “lipid metabolic process”, “response to endoplasmic reticulum stress”, “regulation of Cdc42 protein signal transduction” and others (Figures 7A,B and Supplementary Table S14). According to KEGG pathway analysis, we observed several key pathways such as “cholesterol metabolism”, “PI3K-AKT signaling pathway”, “p53 signaling pathway”, “HIF-1 signaling pathway”, “PPAR signaling pathway”, “Ras signaling pathway”, “cell cycle” and “MAPK signaling pathway” (Figure 7E and Supplementary Table S14).

In terms of the DE lncRNAs, GO term analysis showed that the target genes were enriched in “positive regulation of signal transduction by p53 class mediator’, “S-adenosylmethionine-dependent methyltransferase activity”, “cyclic-GMP-AMP binding”, “negative regulation of histone H3-K79 methylation’, “negative regulation of RNA biosynthetic process” and so on (Figures 7C,D and Supplementary Table S15). Although no KEGG pathways were significantly enriched, many target genes were involved in multiple metabolic pathways (Figure 7F and Supplementary Table S15).

### Validation of mRNAs and lncRNAs by qRT-PCR

**FIGURE 8:**
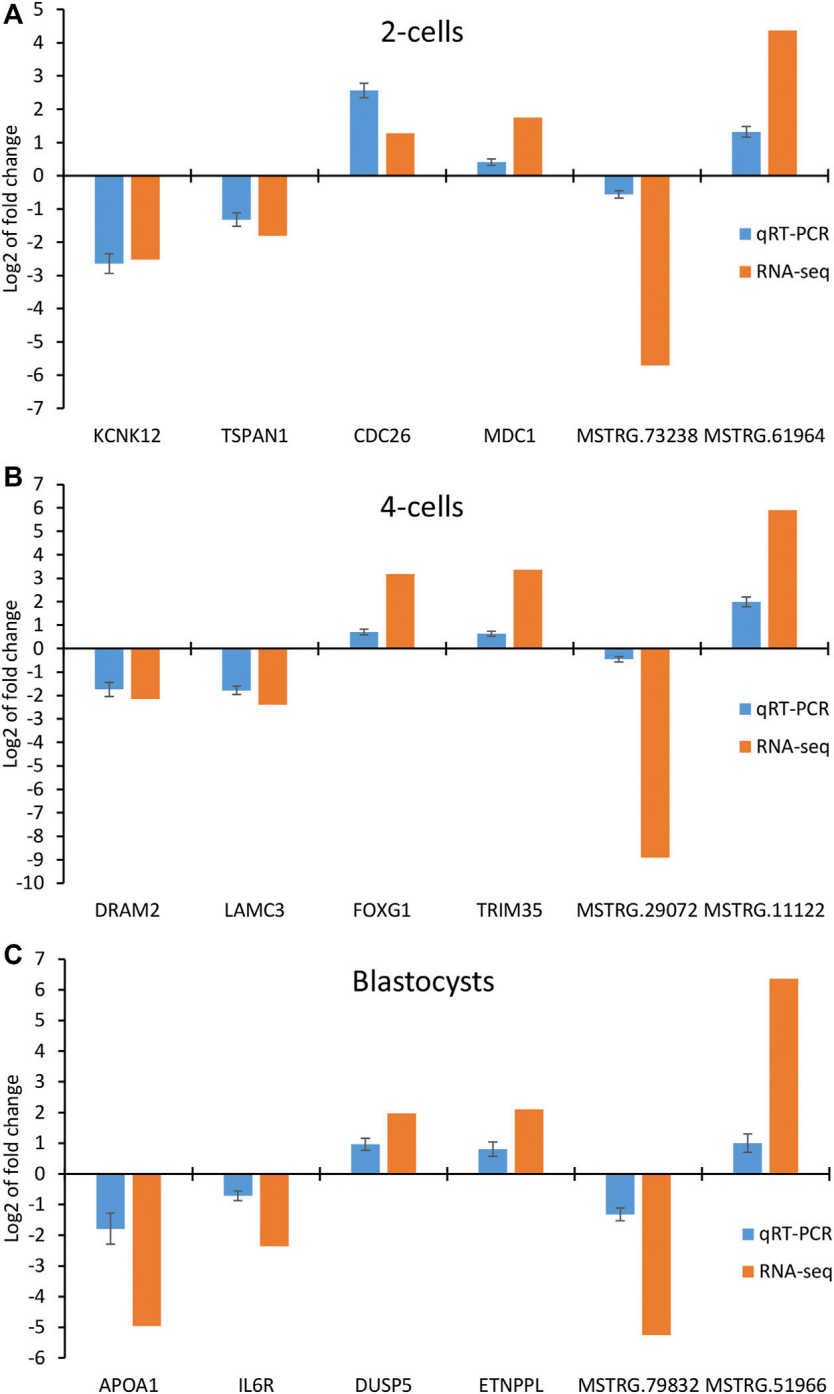
Validation of differentially expressed mRNAs and lncRNAs in **(A)** 2-cells, **(B)** 4-cells and **(C)** blastocysts derived from vitrified porcine cloned zygotes using qRT-PCR.

To further validate the results obtained by RNA-seq, we performed qRT-PCR to detect the expression of 4 mRNAs and 2 lncRNAs selected from 2-cells (*KCNK12*, *CDC26*, *TSPAN1*, *MDC1*, *MSTRG.73238* and *MSTRG.61964*), 4-cells (*DRAM2*, *LAMC3*, *FOXG1*, *TRIM35*, *MSTRG.29072* and *MSTRG.11122*) and blastocysts (*APOA1*, *IL6R*, *DUSP5*, *ETNPPL*, *MSTRG.79832* and *MSTRG.51966*), respectively. As shown in Figures 8A–C, the relative expression fold changes of these genes and lncRNAs in the three embryonic stages were consistent with the RNA-Seq data, suggesting that our transcript results were reliable.

## Discussion

In our previous study, the porcine cloned zygotes after vitrification showed a reduced embryo development *in vitro*, and abnormal expression of mRNA levels was also found in the resultant blastocysts (Jia et al., 2020). Therefore, it is important to continue in-depth research, focusing on the dynamic transcriptome landscapes in different developmental stages of these vitrified zygotes. In the present study, we collected samples from the 2-cells, 4-cells and blastocysts, and acquired extensive mRNA and lncRNA profiles using the RNA-Seq technique. The transcriptomic changes in the three key stages of embryo development would be useful for understanding the cryodamage mechanisms by which vitrification compromised subsequent developmental competence of porcine cloned zygotes.

In this study, we identified 167 DE mRNAs and 516 DE lncRNAs in 2-cells between the groups of vitrified and fresh zygotes. Recent studies have confirmed the abnormal epigenetic modifications in oocytes and embryos after vitrification (Chen et al., 2019; Movahed et al., 2019). Our GO enrichment analysis also found several significantly enriched terms related to epigenetic modifications including “histone H3-K27 trimethylation”, “histone H3-K4 trimethylation” and “histone H3-K27 methylation”. So, the down-regulated expression of *HIST1H1D*, *HIST1H1E* and *EED* genes involved in above terms could be responsible for epigenetic disturbances at 2-cell stage of the vitrified porcine cloned zygotes. We further focused on a GO term of “cellular response to leukemia inhibitory factor”, as it is reported that leukemia inhibitory factor supplemented in the medium can improve blastocyst development and cryotolerance (Kocyigit and Cevik, 2015). Moreover, *GCLM* gene encodes the modifier subunit of glutamate cysteine ligase that is the first-rate limiting enzyme in glutathione biosynthesis (McConnachie et al., 2013), and so the up-regulated expression of this gene may be a response to oxidative stress caused by vitrification. The KEGG pathway analysis revealed that vitrification of porcine cloned zygotes affected some specific pathways in the 2-cells. For example, both “p53 signaling pathway” and “base excision repair” as important stress response pathways have gained a role in the DNA damage response system (Bauer et al., 2015; Du et al., 2016). Their changes indicated that 2-cells from vitrified zygotes presented with cellular senescence, oxidative stress and DNA damage and other conditions. Furthermore, we speculated that “mTOR signaling pathway” and “Hippo signaling pathway” might be associated with the abnormal development of the vitrified zygote-derived 2-cells, because these two pathways are essential for growth and proliferation in embryos (Murakami et al., 2004; Hirate et al., 2013). On the other hand, 11 target genes of DE lncRNAs were significantly enriched in a “defense response” GO term, suggesting 2-cell embryo competence to respond to the impact of vitrification. In addition, no significant pathways were enriched in KEGG analysis, and “RIG-I-like receptor signaling pathway” might play a role in this process.

The present study showed that 469 mRNAs were differentially expressed in the 4-cells derived from vitrified zygotes, which was the largest number among three developmental stages of embryos. According to the GO enrichment results, many DE mRNAs were enriched in “nucleus”, “nucleoplasm”, “nucleic acid binding”, “RNA binding” and “negative regulation of transcription, DNA-templated”, suggesting that vitrification might influence the event of embryo genome activation (EGA) occurring in porcine 4-cells. We further found several stress response-related GO terms caused by vitrification, such as ‘response to oxidative stress”, “response to endoplasmic reticulum stress”, “ER overload response”, “DNA damage response”, “response to hydrogen peroxide” and so on. The *PPP1R15B* gene enriched in these GO terms could be profoundly important, and it is also considered as a EGA marker (Rissi et al., 2019). Endoplasmic reticulum stress has been reported to exert effects in mammalian oocyte maturation and embryo development (Lin et al., 2019), and occurs in the vitrified oocytes and embryos (Barrera et al., 2018; Khatun et al., 2020). Our KEGG analysis also found that ‘protein processing in endoplasmic reticulum’ was a most representative pathway enriched for the up-regulated genes and could be a coping capability to endoplasmic reticulum stress. Moreover, vitrification might not be favorable for the cell cycle progression of 4-cell embryos, based on the GO terms of “negative regulation of G1/S transition of mitotic cell cycle”, “negative regulation of cell cycle” and “negative regulation of mitotic cell cycle”. We also observed the up-regulated genes were enriched in the “cell cycle” pathway, which could possibly be involved in the repair mechanisms to drive the cell cycle properly. In addition, many metabolic pathways were found to be significantly enriched, including “glycerolipid metabolism”, “sphingolipid metabolism”, “lysine degradation”, “fatty acid elongation”, “glycerophospholipid metabolism”, and “beta-alanine metabolism”. As we known, endogenous lipid droplets and exogenous amino acids are important source of metabolic energy for embryos. Therefore, the changes of these pathways and genes related to metabolism might also affect subsequent embryo development. On the other hand, a total of 565 DE lncRNAs were identified in the transcriptomic profiles, and some of these target genes were involved in GO terms such as “G1 to G0 transition”, “embryonic axis specification”, “connexin complex”, “histone H3-K4 trimethylation’, “linoleic acid metabolic process” and others. These terms appeared to be closely associated with embryo development, and so indicated the adverse effects of vitrification on the 4-cell embryos.

A total of 389 mRNAs and 816 lncRNAs were differentially expressed in the blastocysts developed from vitrified cloned zygotes. It is well known that lipid metabolism in the blastocyst stage is a fundamental function needed for subsequent implantation and placentation (Kajdasz et al., 2020). In this study, some GO terms related to lipid function were found to be enriched, such as ‘high-density lipoprotein particle remodeling”, lipoprotein particle”, “negative regulation of very-low-density lipoprotein particle remodeling”, “cholesterol homeostasis”, “phospholipid efflux” and so on, and most of the involved mRNAs showed downregulation. The results were similar with those reported in bovine blastocytes cryopreserved at morula stage (Gupta et al., 2017). Moreover, the down-regulated expression of *APOA1*, *APOC3*, *APOE*, *NR1I3* and *SCARB1* genes also suggested disrupted triglyceride metabolism and lipid-related metabolism, leading to abnormal embryo development. The blastocysts vitrified at the zygote stage still exhibited cellular stresses based on “response to endoplasmic reticulum stress” and “cellular response to reactive oxygen species”. In addition, we found that vitrification influenced the GO terms of ‘positive regulation of cell population proliferation”, “positive regulation of cell migration” and “cell-cell junction”. These terms are tightly associated with the establishment of both trophectoderm and inner cell mass in blastocysts. So, their changes probably led to a slow process of blastocyst formation in the vitrified zygotes as found in our study. According to KEGG pathway enrichment analysis, several important signaling pathways were significantly perturbed following vitrification. For instance, “PI3K-Akt signaling pathway” is an important regulator of cell cycle, and its inhibition results in delayed blastocyst hatching (Zhang et al., 2007). Another example is the “PPAR signaling pathway”, which is involved in cell proliferation and lipid metabolism and has a potential role during embryo development and implantation (Sidrat et al., 2020). Therefore, the altered signaling pathways in blastocysts developed from vitrified zygotes could be a putative cause of unsuccessful pregnancy after embryo transfer.

## Conclusion

To the best of our knowledge, the present study was the first to provide a very detailed transcriptomic analysis for the vitrified porcine cloned zygotes at different stages of embryo development. A large amount of mRNAs and lncRNAs were found to be differentially expressed in the 2-cells (167 mRNAs and 516 lncRNAs), 4-cells (469 mRNAs and 565 lncRNAs) and blastocysts (389 mRNAs and 816 lncRNAs). The function enrichment analysis of DE mRNAs revealed that vitrification influenced many regulatory mechanisms related to cell cycle, cell proliferation, apoptosis, metabolism, etc., Moreover, the target genes of DE lncRNAs could be involved in stress response, metabolism, embryo development and so on. These results suggested that zygote vitrification altered gene expression patterns and biological pathways during the subsequent embryo developmental stages, which might have contributed to a better understanding of the potential cryodamage mechanisms responsible for impaired embryo development, and help to further improve the current vitrification procedures. In addition, the functions of lncRNA have not yet been fully identified in pigs, and so our study will provide valuable information for future related researches.

## Data Availability

The datasets presented in this study can be found in online repositories. The names of the repository/repositories and accession number(s) can be found below: https://www.ncbi.nlm.nih.gov/geo/, GSE180219.

## Supplementary Material

Supplementary Table S1 Primers used for qRT-PCR analysis.

Supplementary Table S2 Overview of RNA-seq data.

Supplementary Table S3 Differentially expressed mRNA in 2-cells.

Supplementary Table S4 Differentially expressed mRNA in 4-cells.

Supplementary Table S5 Differentially expressed mRNA in blastocysts.

Supplementary Table S6 Differentially expressed lncRNA in 2-cells.

Supplementary Table S7 Differentially expressed lncRNA in 4-cells.

Supplementary Table S8 Differentially expressed lncRNA in blastocysts.

Supplementary Table S9 LncRNA target prediction.

Supplementary Table S10 GO and KEGG enrichment analysis of mRNAs in 2-cells.

Supplementary Table S11 GO and KEGG enrichment analysis of lncRNAs in 2-cells.

Supplementary Table S12 GO and KEGG enrichment analysis of mRNAs in 4-cells.

Supplementary Table S13 GO and KEGG enrichment analysis of lncRNAs in 4-cells.

Supplementary Table S14 GO and KEGG enrichment analysis of mRNAs in blastocysts.

Supplementary Table S15 GO and KEGG enrichment analysis of lncRNAs in blastocysts.

## References

[B1] Barrera N. Dos Santos Neto P. C. Cuadro F. Bosolasco D. Mulet A. P. Crispo M. (2018). Impact of Delipidated Estrous Sheep Serum Supplementation on *In Vitro* Maturation, Cryotolerance and Endoplasmic Reticulum Stress Gene Expression of Sheep Oocytes. PLoS One 13, e0198742. 10.1371/journal.pone.0198742 29912910 PMC6005475

[B2] Bartolac L. K. Lowe J. L. Koustas G. Grupen C. G. Sjöblom C. (2018). Effect of Different Penetrating and Non-penetrating Cryoprotectants and media Temperature on the Cryosurvival of Vitrified *In Vitro* Produced Porcine Blastocysts. Anim. Sci. J. 89, 1230–1239. 10.1111/asj.12996 29968319

[B3] Bauer N. C. Corbett A. H. Doetsch P. W. (2015). The Current State of Eukaryotic DNA Base Damage and Repair. Nucleic Acids Res. 43, gkv1136–10101. 10.1093/nar/gkv1136 PMC4666366 26519467

[B4] Beebe L. F. S. Bouwman E. G. McIlfatrick S. M. Nottle M. B. (2011). Piglets Produced from *In Vivo* Blastocysts Vitrified Using the Cryologic Vitrification Method (Solid Surface Vitrification) and a Sealed Storage Container. Theriogenology 75, 1453–1458. 10.1016/j.theriogenology.2010.11.043 21220168

[B5] Bouckenheimer J. Assou S. Riquier S. Hou C. Philippe N. Sansac C. (2016). Long Non-Coding RNAs in Human Early Embryonic Development and Their Potential in ART. Hum. Reprod. Update 23, 19–40. 10.1093/humupd/dmw035 27655590

[B6] Castillo-Martín M. Yeste M. Pericuesta E. Morató R. Gutiérrez-Adán A. Bonet S. (2015). Effects of Vitrification on the Expression of Pluripotency, Apoptotic and Stress Genes in *In Vitro*-Produced Porcine Blastocysts. Reprod. Fertil. Dev. 27, 1072. 10.1071/RD13405 25322142

[B7] Chen H. Zhang L. Wang Z. Chang H. Xie X. Fu L. (2019). Resveratrol Improved the Developmental Potential of Oocytes after Vitrification by Modifying the Epigenetics. Mol. Reprod. Dev. 86, 862–870. 10.1002/mrd.23161 31066155

[B8] Cuello C. Martinez C. A. Nohalez A. Parrilla I. Roca J. Gil M. A. (2016). Effective Vitrification and Warming of Porcine Embryos Using a pH-Stable, Chemically Defined Medium. Sci. Rep. 6, 33915. 10.1038/srep33915 27666294 PMC5036199

[B9] Cuello C. Martinez C. A. Cambra J. M. Parrilla I. Rodriguez-Martinez H. Gil M. A. (2021). Effects of Vitrification on the Blastocyst Gene Expression Profile in a Porcine Model. Int. J. Mol. Sci. 22, 1222. 10.3390/ijms22031222 33513717 PMC7865857

[B10] Deng M. Wan Y. Chen B. Dai X. Liu Z. Yang Y. (2021). Long Non-coding RNA Lnc_3712 Impedes Nuclear Reprogramming via Repressing Kdm5b. Mol. Ther. - Nucleic Acids 24, 54–66. 10.1016/j.omtn.2021.02.016 33738138 PMC7940708

[B11] Du Y. Zhang Y. Li J. Kragh P. M. Kuwayama M. Ieda S. (2007). Simplified Cryopreservation of Porcine Cloned Blastocysts. Cryobiology 54, 181–187. 10.1016/j.cryobiol.2007.01.001 17359960

[B12] Du Z. Zhang Y. Wang G. Peng J. Wang Z. Gao S. (2016). TPhP Exposure Disturbs Carbohydrate Metabolism, Lipid Metabolism, and the DNA Damage Repair System in Zebrafish Liver. Sci. Rep. 6, 21827. 10.1038/srep21827 26898711 PMC4761896

[B13] Ernst E. H. Nielsen J. Ipsen M. B. Villesen P. Lykke-Hartmann K. (2018). Transcriptome Analysis of Long Non-coding RNAs and Genes Encoding Paraspeckle Proteins during Human Ovarian Follicle Development. Front. Cel Dev. Biol. 6, 78. 10.3389/fcell.2018.00078 PMC6066568 30087896

[B14] Esaki R. Ueda H. Kurome M. Hirakawa K. Tomii R. Yoshioka H. (2004). Cryopreservation of Porcine Embryos Derived from In Vitro-Matured Oocytes1. Biol. Reprod. 71, 432–437. 10.1095/biolreprod.103.026542 15044264

[B15] Funahashi H. Cantley T. C. Day B. N. (1997). Synchronization of Meiosis in Porcine Oocytes by Exposure to Dibutyryl Cyclic Adenosine Monophosphate Improves Developmental Competence Following *In Vitro* Fertilization1. Biol. Reprod. 57, 49–53. 10.1095/biolreprod57.1.49 9209079

[B16] Gao L. Jia G. Li A. Ma H. Huang Z. Zhu S. (2017). RNA-seq Transcriptome Profiling of Mouse Oocytes after *In Vitro* Maturation And/or Vitrification. Sci. Rep. 7, 13245. 10.1038/s41598-017-13381-5 29038524 PMC5643491

[B17] Gomis J. Cuello C. Sanchez-Osorio J. Gil M. A. Parrilla I. Angel M. A. (2013). Forskolin Improves the Cryosurvival of In Vivo-derived Porcine Embryos at Very Early Stages Using Two Vitrification Methods. Cryobiology 66, 144–150. 10.1016/j.cryobiol.2012.12.009 23313786

[B18] Gouveia C. Huyser C. Egli D. Pepper M. S. (2020). Lessons Learned from Somatic Cell Nuclear Transfer. Ijms 21, 2314. 10.3390/ijms21072314 PMC7177533 32230814

[B19] Gupta A. Singh J. Dufort I. Robert C. Dias F. C. F. Anzar M. (2017). Transcriptomic Difference in Bovine Blastocysts Following Vitrification and Slow Freezing at Morula Stage. PLoS One 12, e0187268. 10.1371/journal.pone.0187268 29095916 PMC5667772

[B20] Hirate Y. Hirahara S. Inoue K.-i. Suzuki A. Alarcon V. B. Akimoto K. (2013). Polarity-dependent Distribution of Angiomotin Localizes Hippo Signaling in Preimplantation Embryos. Curr. Biol. 23, 1181–1194. 10.1016/j.cub.2013.05.014 23791731 PMC3742369

[B21] Huang J. Ma Y. Wei S. Pan B. Qi Y. Hou Y. (2018). Dynamic Changes in the Global Transcriptome of Bovine Germinal Vesicle Oocytes after Vitrification Followed by *In Vitro* Maturation. Reprod. Fertil. Dev. 30, 1298. 10.1071/RD17535 29661269

[B22] Jia B.-Y. Xiang D.-C. Quan G.-B. Zhang B. Shao Q.-Y. Hong Q.-H. (2019). Transcriptome Analysis of Porcine Immature Oocytes and Surrounding Cumulus Cells after Vitrification and *In Vitro* Maturation. Theriogenology 134, 90–97. 10.1016/j.theriogenology.2019.05.019 31158735

[B23] Jia B. Xiang D. Guo J. Jiao D. Quan G. Hong Q. (2020). Successful Vitrification of Early-Stage Porcine Cloned Embryos. Cryobiology 97, 53–59. 10.1016/j.cryobiol.2020.10.009 33065107

[B24] Jiao Y. Gao B. Wang G. Li H. Ahmed J. Z. Zhang D. (2020). The Key Long Non‐Coding RNA Screening and Validation between Germinal Vesicle and Metaphase II of Porcine Oocyte *In Vitro* Maturation. Reprod. Dom Anim. 55, 351–363. 10.1111/rda.13620 31903647

[B25] Jin B. Higashiyama R.-i. Nakata Y.-i. Yonezawa J.-i. Xu S. Miyake M. (2013). Rapid Movement of Water and Cryoprotectants in Pig Expanded Blastocysts via Channel Processes: Its Relevance to Their Higher Tolerance to Cryopreservation1. Biol. Reprod. 89, 87. 10.1095/biolreprod.112.107250 23966318

[B26] Kajdasz A. Warzych E. Derebecka N. Madeja Z. E. Lechniak D. Wesoly J. (2020). Lipid Stores and Lipid Metabolism Associated Gene Expression in Porcine and Bovine Parthenogenetic Embryos Revealed by Fluorescent Staining and RNA-Seq. Int. J. Mol. Sci. 21, 6488. 10.3390/ijms21186488 PMC7555686 32899450

[B27] Kamoshita M. Kato T. Fujiwara K. Namiki T. Matsumura K. Hyon S.-H. (2017). Successful Vitrification of Pronuclear-Stage Pig Embryos with a Novel Cryoprotective Agent, Carboxylated ε-poly-L-lysine. PLoS One 12, e0176711. 10.1371/journal.pone.0176711 28448636 PMC5407792

[B28] Kawakami M. Kato Y. Tsunoda Y. (2008). The Effects of Time of First Cleavage, Developmental Stage, and Delipidation of Nuclear-Transferred Porcine Blastocysts on Survival Following Vitrification. Anim. Reprod. Sci. 106, 402–411. 10.1016/j.anireprosci.2007.06.002 17628361

[B29] Keefer C. L. (2015). Artificial Cloning of Domestic Animals. Proc. Natl. Acad. Sci. USA 112, 8874–8878. 10.1073/pnas.1501718112 26195770 PMC4517265

[B30] Khatun H. Ihara Y. Takakura K. Egashira J. Wada Y. Konno T. (2020). Role of Endoplasmic Reticulum Stress on Developmental Competency and Cryo-Tolerance in Bovine Embryos. Theriogenology 142, 131–137. 10.1016/j.theriogenology.2019.09.042 31593880

[B31] Kocyigit A. Cevik M. (2015). Effects of Leukemia Inhibitory Factor and Insulin-like Growth Factor-I on the Cell Allocation and Cryotolerance of Bovine Blastocysts. Cryobiology 71, 64–69. 10.1016/j.cryobiol.2015.05.068 26025880

[B32] Lin T. Lee J. Kang J. Shin H. Lee J. Jin D. (2019). Endoplasmic Reticulum (ER) Stress and Unfolded Protein Response (UPR) in Mammalian Oocyte Maturation and Preimplantation Embryo Development. Int. J. Mol. Sci. 20, 409. 10.3390/ijms20020409 PMC6359168 30669355

[B33] McConnachie L. A. Botta D. White C. C. Weldy C. S. Wilkerson H.-W. Yu J. (2013). The Glutathione Synthesis Gene Gclm Modulates Amphiphilic Polymer-Coated CdSe/ZnS Quantum Dot-Induced Lung Inflammation in Mice. PLoS One 8, e64165. 10.1371/journal.pone.0064165 23724032 PMC3664581

[B34] Misumi K. Hirayama Y. Egawa S. Yamashita S. Hoshi H. Imai K. (2013). Successful Production of Piglets Derived from Expanded Blastocysts Vitrified Using a Micro Volume Air Cooling Method without Direct Exposure to Liquid Nitrogen. J. Reprod. Dev. 59, 520–524. 10.1262/jrd.2013-045 23955236 PMC3934155

[B35] Mito T. Yoshioka K. Noguchi M. Yamashita S. Misumi K. Hoshi T. (2015). Birth of Piglets from In Vitro-Produced Porcine Blastocysts Vitrified and Warmed in a Chemically Defined Medium. Theriogenology 84, 1314–1320. 10.1016/j.theriogenology.2015.06.024 26255223

[B36] Movahed E. Soleimani M. Hosseini S. Akbari Sene A. Salehi M. (2019). Aberrant Expression of miR‐29a/29b and Methylation Level of Mouse Embryos after *In Vitro* Fertilization and Vitrification at Two‐cell Stage. J. Cel. Physiol. 234, 18942–18950. 10.1002/jcp.28534 30916357

[B37] Murakami M. Ichisaka T. Maeda M. Oshiro N. Hara K. Edenhofer F. (2004). mTOR Is Essential for Growth and Proliferation in Early Mouse Embryos and Embryonic Stem Cells. Mol. Cel. Biol. 24, 6710–6718. 10.1128/MCB.24.15.6710-6718.2004 PMC444840 15254238

[B38] Nakano K. Matsunari H. Nakayama N. Ogawa B. Kurome M. Takahashi M. (2011). Cloned Porcine Embryos Can Maintain Developmental Ability after Cryopreservation at the Morula Stage. J. Reprod. Dev. 57, 312–316. 10.1262/jrd.10-142a 21242653

[B39] Nguyen V. K. Vu H. T. T. Nguyen H. T. Quan H. X. Pham L. D. Kikuchi K. (2018). Comparison of the Microdrop and Minimum Volume Cooling Methods for Vitrification of Porcine *In Vitro*-produced Zygotes and Blastocysts after Equilibration in Low Concentrations of Cryoprotectant Agents. J. Reprod. Dev. 64, 457–462. 10.1262/jrd.2018-047 30101829 PMC6189571

[B40] Nohalez A. Martinez C. A. Parrilla I. Maside C. Roca J. Gil M. A. (2018). Eventual Re-vitrification or Storage in Liquid Nitrogen Vapor Does Not Jeopardize the Practical Handling and Transport of Vitrified Pig Embryos. Theriogenology 113, 229–236. 10.1016/j.theriogenology.2018.03.001 29567383

[B41] Picelli S. Faridani O. R. Björklund Å. K. Winberg G. Sagasser S. Sandberg R. (2014). Full-length RNA-Seq from Single Cells Using Smart-Seq2. Nat. Protoc. 9, 171–181. 10.1038/nprot.2014.006 24385147

[B42] Rissi V. B. Glanzner W. G. De Macedo M. P. Gutierrez K. Baldassarre H. Gonçalves P. B. D. (2019). The Histone Lysine Demethylase KDM7A Is Required for normal Development and First Cell Lineage Specification in Porcine Embryos. Epigenetics 14, 1088–1101. 10.1080/15592294.2019.1633864 31216927 PMC6773414

[B43] Sidrat T. Khan A. A. Idrees M. Joo M.-D. Xu L. Lee K.-L. (2020). Role of Wnt Signaling during *In-Vitro* Bovine Blastocyst Development and Maturation in Synergism with PPARδ Signaling. Cells 9, 923. 10.3390/cells9040923 PMC7226827 32283810

[B44] Somfai T. Ozawa M. Noguchi J. Kaneko H. Nakai M. Maedomari N. (2009). Live Piglets Derived from In Vitro-Produced Zygotes Vitrified at the Pronuclear Stage1. Biol. Reprod. 80, 42–49. 10.1095/biolreprod.108.070235 18768913

[B45] Svoboda P. (2017). Long and Small Noncoding RNAs during Oocyte-To-Embryo Transition in Mammals. Biochem. Soc. Trans. 45, 1117–1124. 10.1042/BST20170033 28939692

[B46] Tajima S. Uchikura K. Kurita T. Kikuchi K. (2020). Insemination of Recipient Sows Improves the Survival to Term of Vitrified and Warmed Porcine Expanded Blastocysts Transferred Non‐surgically. Anim. Sci. J. 91, e13453. 10.1111/asj.13453 32926526 PMC7539913

[B47] Tang Z. Li Y. Wan P. Li X. Zhao S. Liu B. (2007). LongSAGE Analysis of Skeletal Muscle at Three Prenatal Stages in Tongcheng and Landrace Pigs. Genome Biol. 8, R115. 10.1186/gb-2007-8-6-r115 17573972 PMC2394763

[B48] Trovero M. F. Rodríguez-Casuriaga R. Romeo C. Santiñaque F. F. François M. Folle G. A. (2020). Revealing Stage-specific Expression Patterns of Long Noncoding RNAs along Mouse Spermatogenesis. RNA Biol. 17, 350–365. 10.1080/15476286.2019.1700332 31869276 PMC6999611

[B49] Vining L. M. Zak L. J. Harvey S. C. Harvey K. E. (2021). The Role of Apoptosis in Cryopreserved Animal Oocytes and Embryos. Theriogenology 173, 93–101. 10.1016/j.theriogenology.2021.07.017 34365139

[B50] Wang N. Li C.-Y. Zhu H.-B. Hao H.-S. Wang H.-Y. Yan C.-L. (2017). Effect of Vitrification on the mRNA Transcriptome of Bovine Oocytes. Reprod. Dom Anim. 52, 531–541. 10.1111/rda.12942 28295644

[B51] Wang J. Koganti P. P. Yao J. (2020). Systematic Identification of Long Intergenic Non-Coding RNAs Expressed in Bovine Oocytes. Reprod. Biol. Endocrinol. 18, 13. 10.1186/s12958-020-00573-4 32085734 PMC7035783

[B52] Wang J.-J. Niu M.-H. Zhang T. Shen W. Cao H.-G. (2020). Genome-Wide Network of lncRNA-mRNA during Ovine Oocyte Development from Germinal Vesicle to Metaphase II *In Vitro* . Front. Physiol. 11, 1019. 10.3389/fphys.2020.01019 32973554 PMC7461901

[B53] Wei H. Qing Y. Pan W. Zhao H. Li H. Cheng W. (2013). Comparison of the Efficiency of Banna Miniature Inbred Pig Somatic Cell Nuclear Transfer Among Different Donor Cells. PLoS One 8, e57728. 10.1371/journal.pone.0057728 23469059 PMC3585185

[B54] Whitelaw C. B. A. Sheets T. P. Lillico S. G. Telugu B. P. (2016). Engineering Large Animal Models of Human Disease. J. Pathol. 238, 247–256. 10.1002/path.4648 26414877 PMC4737318

[B55] Wilkinson S. Lu Z. H. Megens H.-J. Archibald A. L. Haley C. Jackson I. J. (2013). Signatures of Diversifying Selection in European Pig Breeds. Plos Genet. 9, e1003453. 10.1371/journal.pgen.1003453 23637623 PMC3636142

[B56] Wu G.-Q. Quan G.-B. Shao Q.-Y. Lv C.-R. Jiang Y.-T. Zhao Z.-Y. (2016). Cryotop Vitrification of Porcine Parthenogenetic Embryos at the Early Developmental Stages. Theriogenology 85, 434–440. 10.1016/j.theriogenology.2015.09.015 26462660

[B57] Xiang D. C. Jia B. Y. Quan G. B. Zhang B. Shao Q. Y. Zhao Z. Y. (2019). Effect of Knockout Serum Replacement during Postwarming Recovery Culture on the Development and Quality of Vitrified Parthenogenetic Porcine Blastocysts. Biopreserv. Biobank. 17, 342–351. 10.1089/bio.2018.0132 31009253

[B58] Yang H. Wu Z. (2018). Genome Editing of Pigs for Agriculture and Biomedicine. Front. Genet. 9, 360. 10.3389/fgene.2018.00360 30233645 PMC6131568

[B59] Yang C. X. Wang P. C. Liu S. Miao J. K. Liu X. M. Miao Y. L. (2020). Long Noncoding RNA 2193 Regulates Meiosis through Global Epigenetic Modification and Cytoskeleton Organization in Pig Oocytes. J. Cel. Physiol. 235, 8304–8318. 10.1002/jcp.29675 32239703

[B60] Yang C. X. Wu Z. W. Liu X. M. Liang H. Gao Z. R. Wang Y. (2021). Single‐cell RNA‐seq Reveals mRNAs and lncRNAs Important for Oocytes *In Vitro* Matured in Pigs. Reprod. Dom Anim. 56, 642–657. 10.1111/rda.13901 33496347

[B61] Yoshioka K. Suzuki C. Tanaka A. Anas I. M.-K. Iwamura S. (2002). Birth of Piglets Derived from Porcine Zygotes Cultured in a Chemically Defined Medium1. Biol. Reprod. 66, 112–119. 10.1095/biolreprod66.1.112 11751272

[B62] Yue Y. Xu W. Kan Y. Zhao H.-Y. Zhou Y. Song X. (2021). Extensive Germline Genome Engineering in Pigs. Nat. Biomed. Eng. 5, 134–143. 10.1038/s41551-020-00613-9 32958897

[B63] Zhang Y. Yang Z. Wu J. (2007). Signaling Pathways and Preimplantation Development of Mammalian Embryos. FEBS J. 274, 4349–4359. 10.1111/j.1742-4658.2007.05980.x 17680809

[B64] Zhang F. Zhang Z.-Y. Cai M.-D. Li X.-X. Li Y.-H. Lei Y. (2020). Effect of Vitrification Temperature and Cryoprotectant Concentrations on the mRNA Transcriptome of Bovine Mature Oocytes after Vitrifying at Immature Stage. Theriogenology 148, 225–235. 10.1016/j.theriogenology.2019.11.006 31761539

[B65] Zhang L. Qi X. Ning W. Shentu L. Guo T. Zhang X. (2020). Single-cell Transcriptome Profiling Revealed that Vitrification of Somatic Cloned Porcine Blastocysts Causes Substantial Perturbations in Gene Expression. Front. Genet. 11, 640. 10.3389/fgene.2020.00640 32793277 PMC7394247

