# Supplementary material for: Transcriptome Analysis of mRNAs and Long Non-Coding RNAs During Subsequent Embryo Development of Porcine Cloned Zygotes After Vitrification
Source: Front Genet. 2021 Dec 17;12:753327. doi: 10.3389/fgene.2021.753327 (PMC8718616; doi:10.3389/fgene.2021.753327)
Supplement: Supplementary file 4 [file Table1.DOCX]

Table S1. Primers used for qRT-PCR analysis.

| **Transcript type** | **Gene ID** | **Gene**  **symbol** | **Primer sequence (5'—3')** | **Fragment**  **Size (bp)** | **GenBank Access No.** |
| --- | --- | --- | --- | --- | --- |
|  |  |  |  |  |  |
| mRNA | ENSSSCG00000001362 | MDC1 | F:5-AGTGATTCCAGAACCAGGCA-3  R:5-CTCTTGTGCAGGTTTGGTCC-3 | 100 | XM_013977588.2 |
| mRNA | ENSSSCG00000033177 | CDC26 | F:5-AATGCCGGATCCTTAACCCA-3  R:5-AGTGGTCTCTTCATTGCCCA-3 | 100 | XM_013993766.2 |
| mRNA | ENSSSCG00000008427 | KCNK12 | F:5-TAGAACAGCCGCTACACACA-3  R:5-AAAGCCTGCTAAGCCCCTTA-3 | 127 | XM_005674606.3 |
| mRNA | ENSSSCG00000010341 | TSPAN1 | F:5-AAATAAGTCTGCCTCCGCCT-3  R:5-ATTAGATCCCTCCCCACCCT-3 | 106 | XM_005665450.3 |
| mRNA | ENSSSCG00000033325 | TRIM35 | F:5-TGCAGAAGGGAGGGAAAGAG-3  R:5-GACAGAACAAGAGGGAGGCT-3 | 145 | XM_001928380.5 |
| mRNA | ENSSSCG00000033761 | FOXG1 | F:5-CCCTGCCCTGTGAGTCTTTA-3  R:5-GGTTGGAAGAAGACCCCTGA-3 | 108 | XM_021099188.1 |
| mRNA | ENSSSCG00000005710 | LAMC3 | F:5-CCCCGTCCTGTGTGAATACT-3  R:5-TGTGTGTGTGTGTATGGGGT-3 | 150 | XM_003353687.5 |
| mRNA | ENSSSCG00000026922 | DRAM2 | F:5-TGTCCAGTCCTGATTGCTGT-3  R:5-ATCAAGTCCCTGTTCCTCCC-3 | 150 | XM_005663559.3 |
| mRNA | ENSSSCG00000009144 | ETNPPL | F:5-TCTTCTGCTGTTGGTTTGGC-3  R:5-AGGCCAACACCCCTGATATC-3 | 149 | XM_021101686.1 |
| mRNA | ENSSSCG00000040815 | DUSP5 | F:5-AGGTCCTTGTCCACTGTGAG-3  R:5-CACACTCCTCCTCTGCTTGA-3 | 125 | XM_003359366.4 |
| mRNA | ENSSSCG00000030921 | APOA1 | F:5-CTGGGATCGGGTGAAGGATT-3  R:5-TGAGGTGTTTTCCCAAAGCG-3 | 107 | NM_214398.1 |
| mRNA | ENSSSCG00000027628 | IL6R | F:5-ACCCTAAGCTTTCCCTGTCC-3  R:5-GGTATTATTGGCCGAGCTGC-3 | 134 | XM_021088726.1 |
| mRNA |  | GAPDH | F:5-AAGTTCCACGGCACAGTCAAG-3  R:5-CACCAGCATCACCCCATTT-3 | 112 | AK234838 |
| lncRNA | MSTRG.73238 | MSTRG.73238.1 | F:5-TTCGGTGTGCTGATGACAAT-3  R:5-CGTTTGCTCGGATTTGACTT-3 | 131 |  |
| lncRNA | MSTRG.61964 | MSTRG.61964.1 | F:5-AAGTGGGTTGGTGTGAAAGC-3  R:5-GGGACACAGACTGGTGAGGT-3 | 126 |  |
| lncRNA | MSTRG.11122 | MSTRG.11122.4 | F:5-CCCTTGATTGTCCTCGTGAT-3  R:5-CAAAGCCTTGCCAAAAAGAG-3 | 103 |  |
| lncRNA | MSTRG.29072 | MSTRG.29072.11 | F:5-ACAGTGAGACGGGCTGAACT-3  R:5-GTGCCGTTGTTTGGTTTTCT-3 | 94 |  |
| lncRNA | MSTRG.51966 | MSTRG.51966.7 | F:5-TCCGACTAGGAACCATGAGG-3  R:5-TCTGCAACCTACACCACAGC-3 | 96 |  |
| lncRNA | MSTRG.79832 | MSTRG.79832.3 | F:5-TCCGACTAGGAACCATGAGG-3  R:5-TCTGCAACCTACACCACAGC-3 | 96 |  |
